# Adolescent intermittent ethanol exposure induces sex-specific and time-dependent changes in affective behaviors and metabolomic profiles

**DOI:** 10.3389/fnbeh.2025.1614537

**Published:** 2026-01-30

**Authors:** Mariah J. Shobande, Anjali Kumari, Michael Pearson, Janae A. Baker, Nzia I. Hall, Renee C. Waters, Chloe E. Emehel, Dashear Hill, Myla E. Fowlkes, Tiffany Dean, Reginald Cannady, Bo Wang, Antoniette M. Maldonado-Devincci

**Affiliations:** 1 Department of Chemical, Biological and Bioengineering, College of Engineering, North Carolina Agricultural and Technical State University, Greensboro, NC, United States; 2 Department of Biology, College of Science and Technology, North Carolina Agricultural and Technical State University, Greensboro, NC, United States; 3 Department of Social Work and Sociology, Hairston College of Health and Human Sciences, North Carolina Agricultural and Technical State University, Greensboro, NC, United States; 4 Department of Psychology, College of Health and Human Sciences, North Carolina Agricultural and Technical State University, Greensboro, NC, United States; 5 University of North Carolina at Chapel Hill School of Medicine, Chapel Hill, NC, United States; 6 Department of Psychology, Princeton Neuroscience Institute, Princeton University, Princeton, NJ, United States; 7 Department of Chemistry and Chemical Engineering, Florida Institute of Technology, Melbourne, FL, United States; 8 Department of Biomedical and Chemical Engineering and Sciences, Florida Institute of Technology, Melbourne, FL, United States

**Keywords:** adolescence, AIE, alcohol, amino acids, binge-like ethanol exposure, fecal samples, liver, metabolomics

## Abstract

Adolescent binge drinking in humans is associated with adverse outcomes, here we examined sex- and withdrawal-dependent changes in affective behaviors and metabolomic profiles in male and female mice following AIE exposure. Male and female C57BL/6 J mice were exposed to intermittent ethanol vapor inhalation from postnatal (PND) 28–42, a model of intermittent binge-like ethanol exposure during adolescence. Affective behavior was assessed using the open field test (OFT), light/dark test (LDT), and tail suspension test (TST) during short-term withdrawal (PND 49–53) and again during long-term withdrawal (PND 91–95). Serum samples were collected 24 h after the final exposure cycle (PND 43), fecal samples were collected during each OFT, and liver samples were collected at euthanasia (PND 119; ~80 days after exposure) for metabolomic analysis. Ethanol sensitivity in adulthood was additionally assessed using loss of righting reflex (LORR) on PND 116. Overall, AIE produced modest, sex- and withdrawal-dependent behavioral effects, whereas metabolomic differences were most pronounced in males shortly after exposure and diminished with longer withdrawal. These findings support future work testing whether early metabolomic shifts track later behavioral vulnerability.

## 1 Introduction

Adolescents commonly engage in high-risk behaviors, including alcohol use. Specifically, in 2019 among 16–17 year-old teens, 17.8% of boys and 20.8% of girls reported using alcohol in the past month (SAMHSA, 2020). While adolescents drink less frequently than adults, when they do drink, binge drinking is the most common pattern of consumption (Windle, 2016), with 10.2% of boys and 11.2% of girls reporting binge drinking in the 2019 National Survey on Drug Use and Health (SAMHSA, 2020). Over the past several years, sex differences in binge drinking rates in teens have narrowed, and teenage girls are drinking similar or more alcohol compared to teenage boys (SAMHSA, 2020). This early profile in alcohol drinking rates during adolescence sets the stage for a higher percentage of adult women to develop health-related complications faster than men, despite lower overall lifetime alcohol consumption (Fama et al., 2020; White, 2020).

Heavy alcohol exposure can alter physical, cognitive, social, emotional, behavioral, and neural development during adolescence (Hiller-Sturmhöfel and Spear, 2018; Spear and Swartzwelder, 2014; Thorpe et al., 2020; Marco et al., 2017). Studies in animal models, mostly in rats, show persistent effects of adolescent intermittent ethanol (AIE) exposure in both sexes, with effects that are sex- and task-dependent, including ethanol drinking, anxiety-like behavior, impulsivity, behavioral flexibility, memory, sleep, and social anxiety (Crews et al., 2019; Dannenhoffer et al., 2018; Pandey et al., 2015; Robinson et al., 2021; Sakharkar et al., 2019; Healey et al., 2022). Together, clinical data and preclinical studies in both sexes support the conclusion that binge-like ethanol exposure during adolescence can increase risk for affective dysregulation, though effects vary by task and withdrawal timepoint (Dannenhoffer et al., 2018; Gilpin, 2012; Pandey et al., 2015; Varlinskaya et al., 2017). However, most research to date investigating AIE effects has focused on alterations in neurobehavioral functioning, and little work in animal models has included analyses of AIE-induced changes in peripheral physiology, including metabolic and gut-related processes.

Sex differences in the acute and long-term effects of adolescent ethanol exposure are increasingly found in clinical and preclinical research. In rats, AIE exposure sex-specifically alters affective behaviors including exploratory and anxiety-like behaviors (Varlinskaya et al., 2020; Aguilar et al., 2023; Healey et al., 2022), fear conditioning (Chandler et al., 2022), and social drinking (Towner et al., 2022) in adulthood. We recently observed sex differences following withdrawal from AIE and voluntary alcohol drinking; with females showing changes in anxiety-like behavior during short-term and long-term withdrawal; whereas male AIE-exposed mice showed changes in anxiety-like behavior (Maldonado-Devincci et al., 2022) and stress reactivity (Healey et al., 2023) only after long-term withdrawal. Other work shows greater AIE-induced changes in affective behaviors (e.g., novelty-induced hypophagia) in females compared to male C57BL/6 J mice (Kasten et al., 2020). We also recently showed that AIE induced long-term increases in Grin2b in the cerebellum following long-term withdrawal in both male and female mice (Healey et al., 2023). Together, these data indicate that AIE alters later behavioral changes in a sex-specific and withdrawal time-dependent manner, but more work is needed to understand the underlying long-lasting biological changes that mediate these behavioral alterations, including differences in alcohol pharmacokinetics and alcohol metabolism seen between sexes (Kezer et al., 2021).

Metabolomics is a useful scientific approach for analyzing minimally invasive samples to determine which biochemical processes may be altered in the body due to certain stimuli (Costanzo et al., 2022). Metabolomics can be a powerful tool to complement other ‘omics approaches to indicate the function of an organism and to identify specific biomarkers that may be altered following different environmental perturbations (Harrigan et al., 2008). In preclinical work, metabolomic responses to environmental challenges can also be sex-dependent and sample type–dependent. For example, we recently showed that high-fat diet exposure in C57BL/6 J mice produced detectable, sex-specific metabolite shifts in both fecal and liver samples, with differences in glycolytic/energy-related metabolites and amino acid–related profiles that varied by sex and tissue (Wang B. et al., 2023). This approach is used to determine molecular phenotypes of various diseases and disorders like diabetes, cardiovascular disease, liver disease, changes in affective behavior, and alcohol and substance use disorders (Tilg et al., 2016; Williams et al., 2014; Mostafa et al., 2017; Ghanbari et al., 2021; Voutilainen and Kärkkäinen, 2019; Harrigan et al., 2008; Harada et al., 2016). In humans, AUD is characterized by altered metabolomic profiles and leaky gut, along with higher scores on affective measures of anxiety and depression (Leclercq et al., 2014, 2024). Alcohol use is also linked to changes in metabolites including amino acids, lipids, and an increased propensity for liver diseases (Voutilainen and Kärkkäinen, 2019; Tilg et al., 2016; Leclercq et al., 2024). People with AUD were distinguished from social drinkers and nondrinkers using metabolomics and identified differences in propionic acid and acetic acid in plasma (Mostafa et al., 2017). To date, there are few systematic analyses of sex-based differences in metabolomic profiles in humans because of low-powered samples or the lack of systematic data analysis (Costanzo et al., 2022).

Using preclinical models to determine the consequences of alcohol exposure during adolescence is a powerful approach due to ethical constraints that limit observational/survey-based research typically conducted in human studies. Preclinical studies help overcome ethical limits in human research and can serve as proxies for our understanding of alcohol-induced changes in human metabolomics (Humer et al., 2020a; Humer et al., 2020b). Plasma, urine, and fecal samples are minimally invasive sampling methods for assessing alcohol-induced changes in metabolomic profiles (Mostafa et al., 2017; Wang X. et al., 2023). Previous research has used mouse models to determine early biomarkers of alcohol-induced liver disease (Manna et al., 2010; Manna et al., 2015; Manna et al., 2011; Bradford et al., 2008; Charkoftaki et al., 2022). Recently an adult male rat model of AUD linked metabolomic profiles with alcohol drinking and anxiety-like behaviors (Wang X. et al., 2023). However, to date there is limited work using mouse models to determine the link between affective behaviors and changes in metabolomic profiles. Here, AIE vapor exposure models intermittent binge-like ethanol exposure during adolescence, rather than established alcohol dependence or AUD. We therefore examined short-term and long-term changes in metabolomic profiles and affective behaviors in male and female C57BL/6 J mice following AIE exposure. We expected that AIE would produce detectable shifts in metabolomic profiles relative to air-exposed controls across serum, fecal, and liver, and that patterns would differ by sex across these sample types. We also expected sex- and withdrawal-dependent effects of AIE on affect-related behavior across assays, including anxiety-like measures in the open field and light/dark test, and immobility-related behavior in the tail suspension test (TST). Specifically, we expected the timing of detectable behavioral effects to differ by sex (females more evident during short-term withdrawal; males more evident after long-term withdrawal), but we did not predict a uniform directional shift across all behavioral measures given known task- and timepoint-dependent variability in AIE models.

## 2 Methods

### 2.1 Subjects

**Figure 1:**
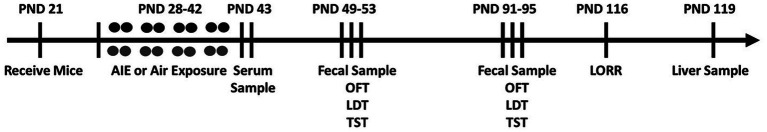
Experimental timeline. Mice were exposed to AIE or AIR between PND 28–42. Serum samples were collected on PND 43. Behavioral testing (OFT, LDT, TST) was conducted over three consecutive days during short-term withdrawal (within PND 49–53) and again during long-term withdrawal (within PND 91–95). Fecal samples were collected during OFT. Mice were challenged with 2.0 g/kg ethanol and assessed for loss of righting reflex (LORR) on PND 116. Livers were collected on PND 119.

Adolescent male and female C57BL/6 J mice (*n* = 10-13/group) were obtained from Jackson Laboratories (Bar Harbor, ME) on PND 21. All mice were allowed to acclimate to the colony for 1 week (PND 21–27) prior to experimentation, where they were handled daily to acclimate them to routine experimenter handling. Mice were PND 28 at the beginning of the experiment. Animals were group-housed (4–5 per cage) with free access to food and water throughout the AIE or AIR exposure (detailed below). An experimental timeline is shown in Figure 1. All mice were maintained in a temperature and humidity-controlled room with lights on from 0700 to 1900 h. Body weights were recorded each time the mice were exposed to the ethanol vapor inhalation chambers and each time they were tested in one of the behavioral tests. Animal care followed National Institutes of Health Guidelines under North Carolina Agricultural and Technical State University Institutional Animal Care and Use Committee approved protocols (21–005).

### 2.2 Adolescent intermittent vapor inhalation chamber exposure

Mice were exposed to repeated intermittent air or ethanol vapor for four two-day exposure cycles from PND 28–42 (Maldonado-Devincci et al., 2022), modeling intermittent binge-like ethanol exposure during adolescence (AIE). On PND 28–29, 32–33, 36–37, 40–41, at approximately 1,630 h, mice were weighed and administered an intraperitoneal injection (0.02 mL/g) of pyrazole (1 mmol/kg), an alcohol dehydrogenase inhibitor used to stabilize blood ethanol concentrations, combined with saline for control mice or combined with 1.6 g/kg ethanol (8% w/v) for AIE-exposed mice and immediately placed in the inhalation chambers (23 in x 23 in x 13 in, Plas Labs, Lansing, MI). Mice remained in the vapor inhalation chambers for 16 h overnight with room (control group) or ethanol (95% ethanol volatilized by passing air through an air stone (gas diffuser) submerged in ethanol) delivered to the chambers at a rate of 10 liters/min. The following morning at 0900 h, mice were removed from the vapor inhalation chamber and 25 μL of blood was collected from the submandibular space for blood ethanol concentration (BEC) assessment and then returned to the home cage for 8 h. On PND 29, 33, 37, and 41, mice were administered pyrazole and placed in the chamber overnight for 16 h. On intervening days, mice remained undisturbed in the home cage. All blood samples were centrifuged at 5,000 *g* and serum was collected and used to analyze blood ethanol concentrations (Table 1) using the AM1 blood alcohol analyzer (Analox Instruments, Lunenburg, MA).

**Table 1:** Blood ethanol concentrations (mg/dL) per cycle in male and female mice exposed to intermittent ethanol vapor inhalation.

Exposure cycles	Male	Female
Cycle 1	283.2 ± 24.1	257.0 ± 5.6
Cycle 2	199.3 ± 19.2	259.4 ± 37.1
Cycle 3	174.0 ± 10.1	191.7 ± 5.6
Cycle 4	180.1 ± 14.9	188.0 ± 8.4

Data are presented as mean ± SEM. Repeated-measures ANOVA results were Geisser–Greenhouse corrected (*ε* = 0.4895).

### 2.3 Behavioral testing

Mice were tested over three consecutive days for behavioral changes using the open field test (OFT), light/dark test (LDT), and tail suspension test (TST) between PND 49–53 (short-term withdrawal) and again between PND 91–95 (long-term withdrawal). For all tests, mice were counterbalanced across days and across groups for testing to minimize between-group differences in testing order. All mice were transported and acclimated to the behavioral testing room for at least 60 min before behavioral testing. Behavioral testing was conducted between 1,000 and 1,600 h during the light phase. All tests were conducted with experimenters blind to the sex and exposure conditions of the mice. Experimental details are described below.

#### 2.3.1 Open field test

Within PND 49–53 and again within PND 91–95, mice were tested for a 60-min session in the open field test (Tatem et al., 2014). Mice were tested with regular overhead lighting (400 lux). Using a Plexiglas chamber (40.6 cm x 40.6 cm), mice were introduced facing one of the corners selected at random to the open field testing arena (Kinder Scientific, Poway, CA) to assess general exploratory behavior (distance traveled and rearing) and activity in the center zone as a measure of anxiety-like behavior (latency, time spent, and distance traveled in the center zone). Immediately upon removal from the arena, mice were returned to their home cage and returned to the colony. The open field arena was cleaned with 70% ethanol and allowed to dry completely before the next mouse was introduced. Data were captured through beam breaks using the SmartFrame Open Field System and quantified using Motor Monitoring Behavioral software (Kinder Scientific, Poway, CA).

#### 2.3.2 Light/dark test

The Light/Dark Test (LDT) is used to evaluate anciety-like behavior in rodents as they have a natural aversion to brightly lit environments (Takao and Miyakawa, 2006). With this test, anxiety in mice can be accurately expressed through their locomotor activity and time spent in each side of the apparatus (Himanshu et al., 2020). During short-term withdrawal (PND 49–53) and long-term withdrawal (PND 91–95), the LDT was conducted for 10 min using the same chamber as the open field test with an infrared transparent black divider (20.3 cm x 20.3 cm) inserted into the dark compartment (Kinder Scientific, Poway, CA). The light side of the box was illuminated with a bright light (850 lux). Mice were introduced to the dark compartment and latency to enter and time spent on the light side were quantified along with distance traveled in both compartments. After each trial, the chamber was sanitized with 70% alcohol and allowed to completely dry. Data were captured through beam breaks using the SmartFrame Open Field System and data were quantified using Motor Monitoring Behavioral software (Kinder Scientific, Poway, CA).

#### 2.3.3 Tail suspension test

The tail suspension test (TST) attempts to translate depressive-like behaviors in humans by observing the initial escape-directed movements made when the rodent is placed in an inescapable environment (Cryan et al., 2005). The TST was conducted within PND 49–53 and again within PND 91–95 to test the longitudinal effects of AIE exposure on depressive-like behavior. The TST was conducted using previously established procedures (Can et al., 2012). After acclimation, mice were briefly restrained to pass the tail through a small plastic cylinder to prevent tail climbing and then attach the adhesive strip to the end of the tail. Mice were then suspended 60 cm above the ground for a duration of 6 min. Mice were then immediately returned to their home cage after the adhesive strip and plastic cylinder were removed. Sessions were recorded and scored offline by experimenters blind to exposure and sex conditions for latency to immobility and duration of immobility. Data were quantified for time spent immobile using previously established protocols (Can et al., 2012).

### 2.4 Loss of righting reflex

On PND 116, all mice were challenged with 2.0 g/kg ethanol and assessed for loss of righting reflex (LORR) according to previously published methods (Silvers et al., 2003; Maldonado-Devincci and Kirstein, 2020). Ethanol challenges and blood collection were conducted during the light phase between 10:00 h and 13:00 h. Briefly, mice received an i.p. injection of ethanol (20% v/v) and were placed in a supine position in a polystyrene reagent reservoir. The duration of LORR was defined as the time until the mouse righted itself on all four paws and maintained that posture for at least 30 s. Mice that did not lose the righting reflex were excluded only from LORR duration analyses (Male-AIR: 1/10; Male-AIE: 7/10; Female-AIR: 2/10; Female-AIE: 5/10) because duration is only interpretable in animals that reach the sedative threshold. These mice were retained for descriptive reporting of LORR incidence (yes/no) and for matched-timepoint BEC confirmation. For mice that did not exhibit LORR, blood ethanol concentration (BEC) samples were collected at a matched post-injection timepoint (when cage mates recovered) to confirm ethanol exposure. For mice that exhibited LORR, blood was collected upon recovery from the submandibular space for BEC measurement. After blood collection, mice were returned to their home cage and allowed to fully recover from intoxication. Mice were left in the home cage until euthanasia on PND 119, when liver samples were collected for metabolite analysis. All blood samples were centrifuged at 5,000 g and serum was collected to analyze BECs using the AM1 Analox Alcohol Analyzer (Analox Instruments, Lunenburg, MA, USA). A subset of recovery BEC samples was lost during processing (Male-AIR: 0/10; Male-AIE: 4/10; Female-AIR: 0/10; Female-AIE: 5/10). No samples were lost for BEC measurements collected after each vapor exposure cycle (Table 1).

### 2.5 Metabolomics

A metabolomic nuclear magnetic resonance (NMR) assay was performed on serum samples collected on PND 43 and fecal samples collected during short-term withdrawal (PND 49–53) and long-term withdrawal (PND 91–95) following the open field test. All samples (serum, fecal, and tissue) were frozen at −80 °C until processed for analysis. Samples were thawed on ice before the NMR analysis. Fecal samples were extracted using water-based on a previously reported method with slight changes (Gratton et al., 2016). These extract samples were mixed with a phosphate buffer of D2O which made the final samples contain 10% of D2O with 0.1 M phosphate buffer (pH = 7.4) and 0.5 mM trimethylsilyl propanoic acid (TSP). The samples were transferred to 5 mm NMR tubes after being centrifuged for further nuclear magnetic resonance (NMR) acquisition.

The serum samples were then extracted using a NaCl approach followed by a previously reported approach. The liver tissue was extracted using a two-step method (Wu et al., 2008) including the homogenization of tissue in cold 2.5:1 methanol–water solvent followed by addition of ice-cold chloroform and water solvent. After centrifugation, the upper polar phase was dried and reconstituted in a phosphate buffer. The final sample phosphate and TSP concentrations were the same as the fecal samples.

### 2.6 Design and analyses

Behavioral data were analyzed using a three-factor mixed-model ANOVA with Exposure (AIR, AIE) and Sex (Male, Female) as between-subjects factors and Withdrawal Time Point (WDT) (short-term withdrawal: within PND 49–53; long-term withdrawal: within PND 91–95) as a within-subjects repeated measure. *A priori* planned exposure effects were assessed within sex for behavioral measures. Blood ethanol concentrations (BECs) during AIR or AIE vapor exposure were analyzed using a two-factor mixed-model ANOVA with Sex as a between-subjects factor and Cycle as a repeated measure. LORR outcomes were analyzed using a two-factor between-subjects ANOVA with Sex and Exposure. Where appropriate, significant effects and/or interactions were followed by Tukey’s HSD or Sidak’s multiple-comparisons tests. Analyses were conducted in GraphPad Prism (v10.4.1).

NMR spectra were preprocessed in Bruker TopSpin 4.11 and reviewed in Bruker AMIX 4.0 prior to peak bucketing. Peak bucketing followed previously established procedures (Wang et al., 2020) with minor modifications. Processed data were normalized to total spectral intensity and exported to Excel (Microsoft). Metabolites were identified using Chenomx 8.4 (Chenomx Inc.). Two-way ANOVAs were computed in Excel, PCA was conducted using PLS-Toolbox (Eigenvector Research), and box plots and heatmaps were generated using MetaboAnalyst 5.0.

## 3 Results

### 3.1 Blood ethanol concentrations

Blood ethanol concentrations (BECs) (Table 1) varied across exposure cycles (Geisser–Greenhouse corrected; *F*(_1.469, 25.95_) = 13.83, *p* = 0.0003, *ε* = 0.4895). There was no main effect of Sex (*F*(_1, 18_) = 0.7304, *p* = 0.4040) and no Cycle × Sex interaction (F(_1.469, 25.95_) = 2.505, *p* = 0.1142), indicating that cycle-related differences did not differ by sex. Sidak’s multiple-comparisons tests showed no male–female differences within any cycle (all *p*adj ≥ 0.4773).

### 3.2 Behavioral assessment

**Figure 2:**
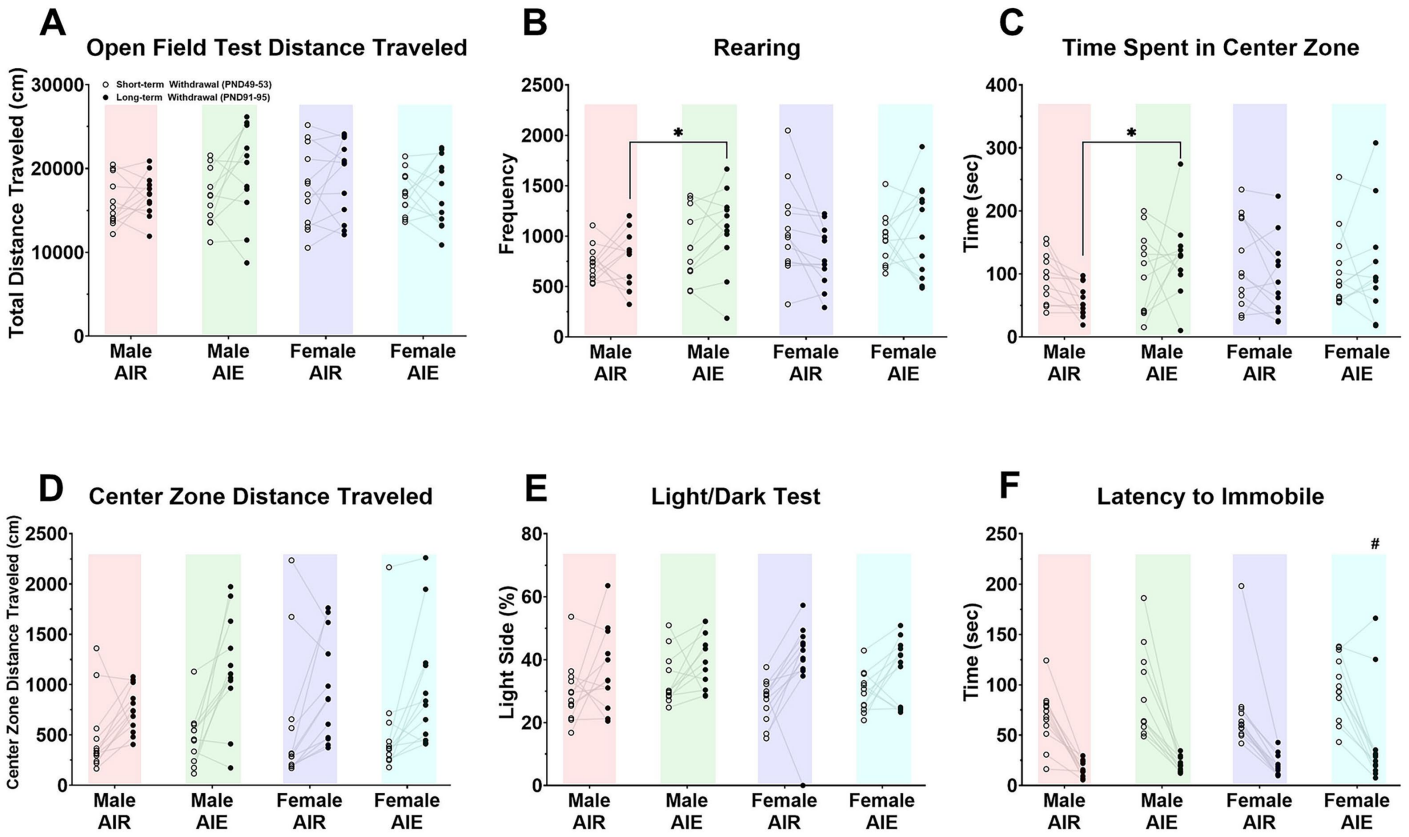
Behavioral measures. Overall behavior was mildly altered after AIE exposure during short-term withdrawal (testing conducted over three consecutive days within PND 49–53) and long-term withdrawal (three consecutive days within PND 91–95). **(A)** Total distance traveled in the open field test (OFT); **(B)** rearing in the open field test; **(C)** time spent in the center zone; **(D)** distance traveled in the center zone; **(E)** percent time spent on the light side of the light/dark test; **(F)** latency to become immobile in the tail suspension test. Data are shown as individual data points. * indicates significant *post hoc* differences (Tukey HSD) between AIR and AIE within sex at the indicated withdrawal timepoint. # indicates a main effect of exposure (AIE vs. AIR). Open circles indicate short-term withdrawal (within PND 49–53) and filled circles indicate long-term withdrawal (within PND 91–95); lines connect repeated measures from the same mouse.

Mice were assessed during short-term withdrawal (testing over three consecutive days within PND 49–53) and again during long-term withdrawal (three consecutive days within PND 91–95). Behavioral testing included the open field test (Figures 2A–D; Tables 2, 3), light/dark test (Figure 2E; Table 4), and tail suspension test (Figure 2F; Table 5).

**Table 2:** Open field test statistics.

Factor	Open field test		Rearing	
	F	*p*	F	*p*
Withdrawal time point (WDT)	F(1,43) = 3.92	0.054	*F*(1,43) = 0.01	0.943
Sex	F(1,43) = 0.27	0.603	F(1,43) = 1.64	0.207
Exposure	F(1,43) = 0.03	0.849	F(1,43) = 3.93	**0.054**
WDT x Sex	F(1,43) = 0.85	0.361	F(1,43) = 2.37	0.131
WDT x exposure	F(1,43) = 0.15	0.696	F(1,43) = 4.72	**0.035**
Sex x exposure	F(1,43) = 2.06	0.159	F(1,43) = 0.78	0.381
WDT x sex x exposure	F(1,43) = 1.26	0.268	F(1,43) = 0.99	0.324

Withdrawal timepoint reflects short-term vs. long-term withdrawal testing. Significant *p*-values are bold.

**Table 3:** Center zone statistics.

Factor	Latency to center zone (*n* = 47)		Center zone time (*n* = 47)		Center zone distance (*n* = 47)		Center zone entries (*n* = 47)	
	F	*p*	F	*p*	F	*p*	F	*p*
Withdrawal time point (WDT)	F(1,43) = 1.11	0.30	F(1,43) = 0.59	0.44	F(1,43) = 33.71	**0.001**	F(1,43) = 0.004	0.95
Sex	F(1,43) = 2.69	0.11	F(1,43) = 0.69	0.41	F(1,43) = 0.13	0.72	F(1,43) = 0.13	0.72
Exposure	F(1,43) = 1.08	0.30	F(1,43) = 2.56	0.11	F (1, 43) = 0.56	0.46	F(1,43) = 1.26	0.27
WDT x sex	F(1,43) = 0.04	0.84	F(1,43) = 0.01	0.98	F(1,43) = 0.31	0.58	F(1,43) = 0.91	0.34
WDT x exposure	F(1,43) = 1.20	0.28	F(1,43) = 4.53	**0.04**	F(1,43) = 2.63	0.11	F(1,43) = 2.06	0.16
Sex x exposure	F(1,43) = 0.02	0.89	F(1,43) = 1.45	0.23	F(1,43) = 0.84	0.37	F(1,43) = 2.10	0.15
WDT x sex x exposure	F(1,43) = 0.01	0.93	F(1,43) = 0.41	0.53	F(1,43) = 1.32	0.26	F(1,43) = 1.56	0.22

Withdrawal timepoint reflects short-term vs. long-term withdrawal testing. Significant *p*-values are bold.

**Table 4:** Light dark test statistics.

Factor	Latency to enter light		Light zone distance (% of total)		Light side entries		Time in light side (%)	
	F	*p*	F	*p*	F	*p*	F	*p*
Withdrawal time point (WDT)	F(1,43) = 0.23	0.64	F(1,43) = 8.88	**0.005**	F(1,43) = 35.68	**0.001**	F(1,43) = 17.22	**<0.001**
Sex	F(1,43) = 9.8	**0.003**	F(1,43) = 0.58	0.45	F(1,43) = 0.99	0.32	F(1,43) = 0.56	0.46
Exposure	F(1,43) = 0.45	0.51	F(1,43) = 0.33	0.57	F(1,43) = 0.28	0.60	F(1,43) = 0.79	0.38
WDT x sex	F(1,43) = 0.41	0.52	F(1,43) = 1.55	0.22	F(1,43) = 0.04	0.84	F(1,43) = 0.68	0.41
WDT x exposure	F(1,43) = 0.02	0.88	F(1,43) = 0.63	0.43	F(1,43) = 0.01	0.94	F(1,43) = 0.68	0.42
sex x exposure	F(1,43) = 0.14	0.71	F(1,43) = 0.28	0.60	F(1,43) = 0.81	0.37	F(1,43) = 0.74	0.40
WDT x sex x exposure	F(1,43) = 0.08	0.79	F(1,43) = 1.55	0.22	F(1,43) = 1.32	0.26	F(1,43) = 0.31	0.59

Withdrawal timepoint reflects short-term vs. long-term withdrawal testing. Significant *p*-values are bold.

**Table 5:** Tail suspension test statistics, withdrawal timepoint reflects short-term vs. long-term withdrawal testing.

Factor	Latency to immobile		Duration immobile	
	F	*p*	F	*p*
Withdrawal time point (WDT)	F (1,43) = 119.4	**<0.001**	F(1,43) = 207.9	**<0.001**
Sex	F (1,43) = 1.44	0.24	F(1,43) = 1.22	*p* = 0.28
Exposure	F (1,43) = 6.44	**0.01**	F(1,43) = 0.86	*p* = 0.36
WDT x sex	F (1,43) = 0.40	0.53	F(1,43) = 0.29	*p* = 0.59
WDT x exposure	F (1,43) = 0.95	0.33	F(1,43) = 0.12	*p* = 0.73
Sex x exposure	F (1,43) = 0.52	0.48	F(1,43) = 0.05	*p* = 0.83
WDT x sex x exposure	F (1,43) = 0.57	0.46	F(1,43) = 0.52	*p* = 0.47

Significant *p*-values are bold.

#### 3.2.1 Open field test

Overall, locomotor activity was not altered by AIE exposure. Total distance traveled in the open field was similar between AIR and AIE groups in both males and females across withdrawal time points (Figure 2A; Table 2).

For exploratory behavior, rearing showed a time-dependent AIE effect in males. Specifically, male mice exhibited greater rearing following AIE relative to AIR controls at long-term withdrawal, whereas males did not differ by exposure at short-term withdrawal. Females showed no exposure-related differences at either withdrawal time point (Figure 2B, Table 2).

For center-zone behavior, there were no differences in latency to enter the center zone, distance traveled in the center zone (Figure 2D), or entries to the center zone (Table 3). However, Male AIE mice spent more time in the center zone compared to Male AIR mice (Figure 2C; Table 3) and this effect appeared to be driven primarily by the long-term withdrawal time point, consistent with the Withdrawal time point × Exposure effect observed for center-zone time (Table 3). In contrast, females did not show exposure-related differences in time spent in the center zone at either withdrawal time point (Figure 2C; Table 3).

#### 3.2.2 Light/dark test

For the light/dark test (LDT), male mice showed a longer latency to enter the light compartment compared to females, independent of exposure condition. In contrast, there were no exposure-related differences in distance traveled in the light compartment, number of entries into the light compartment, or percent time spent in the light compartment (Figure 2E; Table 4).

#### 3.2.3 Tail suspension test

For the tail suspension test, mice became immobile more quickly during long-term withdrawal compared to short-term withdrawal (Figure 2F; Table 5). In female mice, AIE exposure was associated with longer latency to become immobile compared to AIR mice, however, this exposure effect was absent in males (Table 5). There were no exposure-related differences in duration of immobility at either time point; however, both males and females spent more time immobile during long-term withdrawal compared to short-term withdrawal (Table 5).

### 3.3 Metabolomics: metabolite analyses

Supplemental results showing correlations between behavior and specific metabolites are shown in Supplementary material.

#### 3.3.1 Serum metabolites

**Figure 3:**
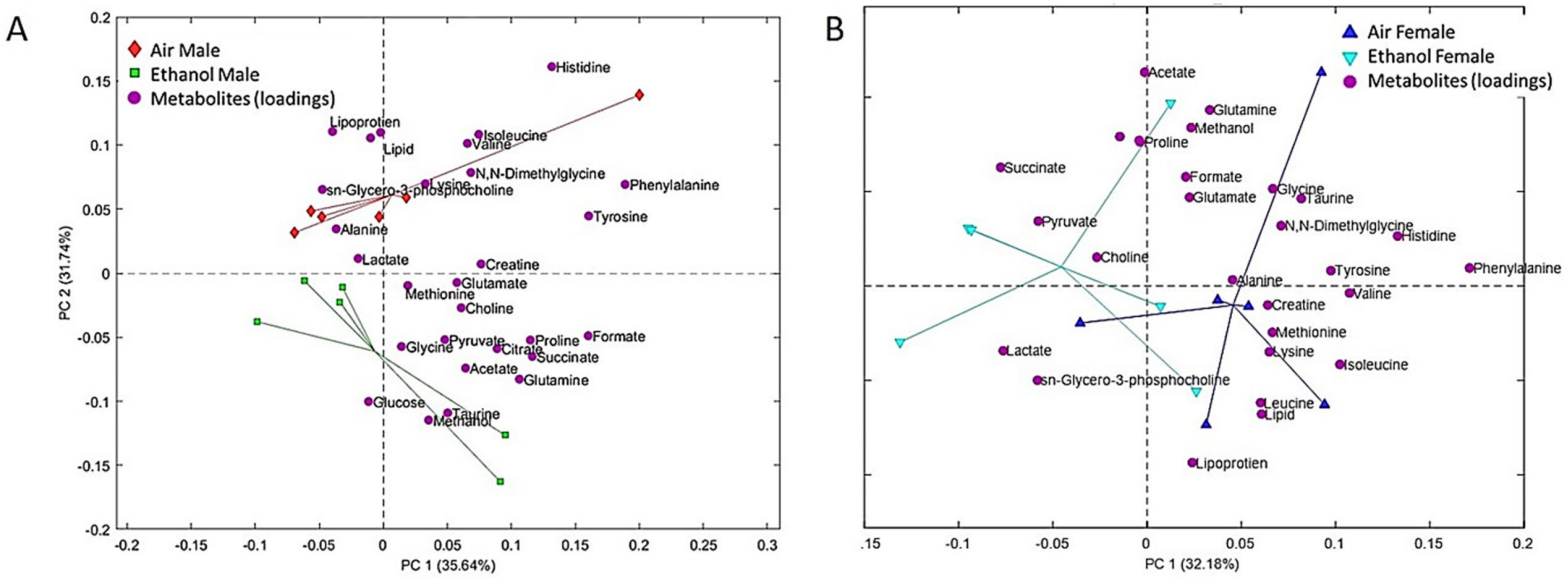
PCA of serum samples. **(A)** PCA results from male serum samples. Red diamonds are male AIR and green squares are male AIE. **(B)** PCA results from female serum samples. Blue upward triangles are female AIR and cyan downward triangles are female AIE. Magenta circles (both panels) represent metabolite loadings.

**Figure 4:**
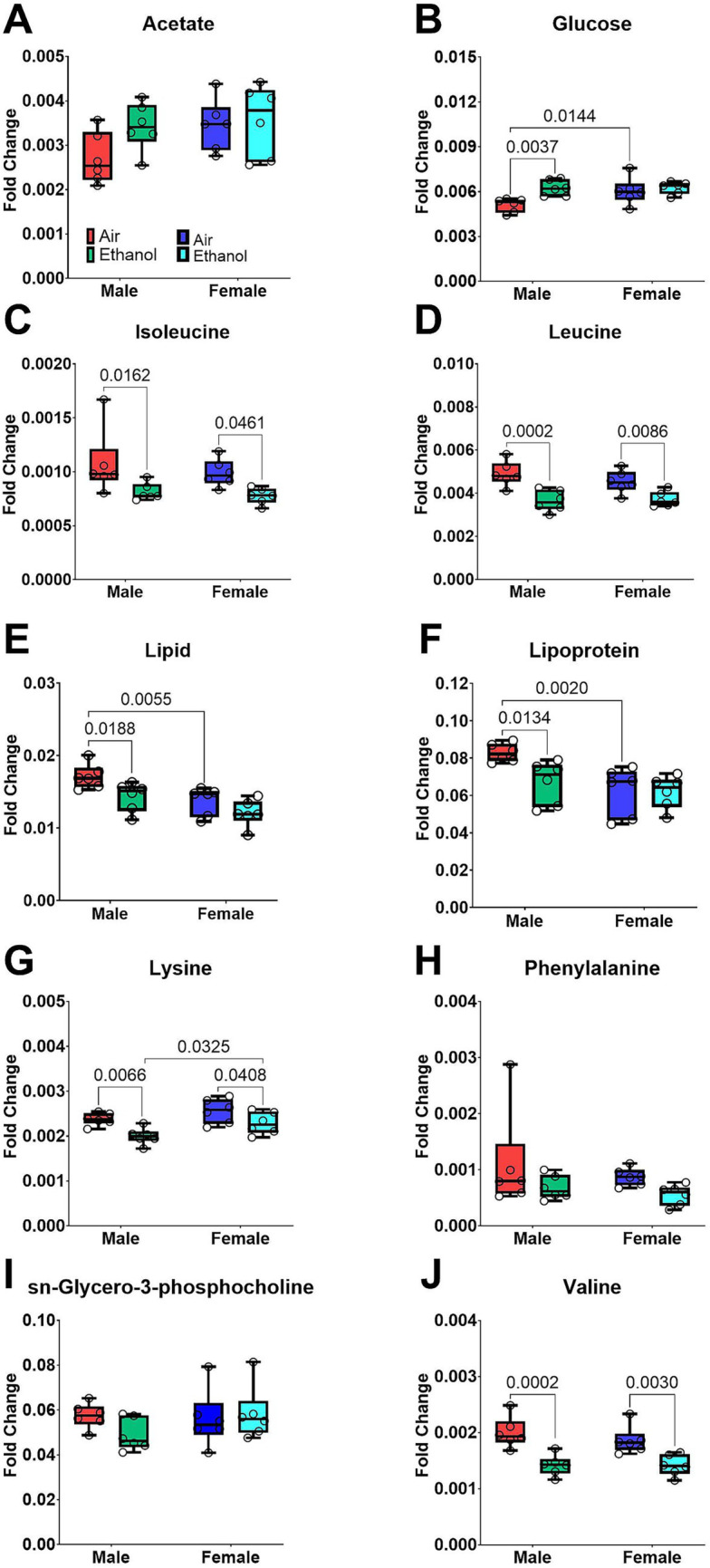
Selected metabolic changes in serum samples collected on PND 43. Samples include male AIR (red), male AIE (green), female AIR (blue), and female AIE (cyan). Data are represented as box-and-whisker plots showing median and range. All data points are shown as open circles. **(A)** Acetate; **(B)** Glucose; **(C)** Isoleucine; **(D)** Leucine; **(E)** Lipid; **(F)** Lipoprotein; **(G)** Lysine; **(H)** Phenylalanine; **(I)** sn-Glycero-3-phosphocholine; **(J)** Valine.

Serum samples were collected 24 h after the last exposure cycle on PND 43 for all mice. The PCA score plot showed better separation among male samples (Figure 3A) than female samples (Figure 3B), indicating that ethanol exposure more strongly influenced serum metabolomic profiles in males. Metabolites showing significant changes after exposure or between sexes are presented in Figures 4A–J. PCA loadings suggested that amino acids, including isoleucine [Exposure (F_1, 20_ = 11.29, *p* = 0.003); Figure 4C], leucine [Exposure (F_1, 20_ = 27.10, *p* < 0.0001); Figure 4D], and valine [Exposure (F_1, 20_ = 30.67, *p* < 0.0001); Figure 4J] contributed substantially to the separation of AIR samples from AIE samples, with all three significantly downregulated after ethanol exposure. These findings indicated reduced circulating levels of amino acids following AIE exposure. Asparagine [Sex by Exposure (F_1, 20_ = 4.53, *p* < 0.05); Figure 4A], glucose [Exposure (F_1, 20_ = 7.71, *p* < 0.0030); Figure 4B], lipid [Exposure (F_1, 20_ = 8.54, *p* < 0.01); Sex (F_1, 20_ = 13.73, *p* < 0.002); Figure 4E] and lipoprotein [Sex (F_1, 20_ = 9.94, *p* < 0.006); Exposure (F_1, 20_ = 3.91, *p* = 0.06); Sex by Exposure (F_1, 20_ = 3.46, *p* = 0.07); Figure 4F] were also affected by AIE exposure, with stronger changes in males and comparatively weaker changes in females.

#### 3.3.2 Fecal metabolites: short term withdrawal

**Figure 5:**
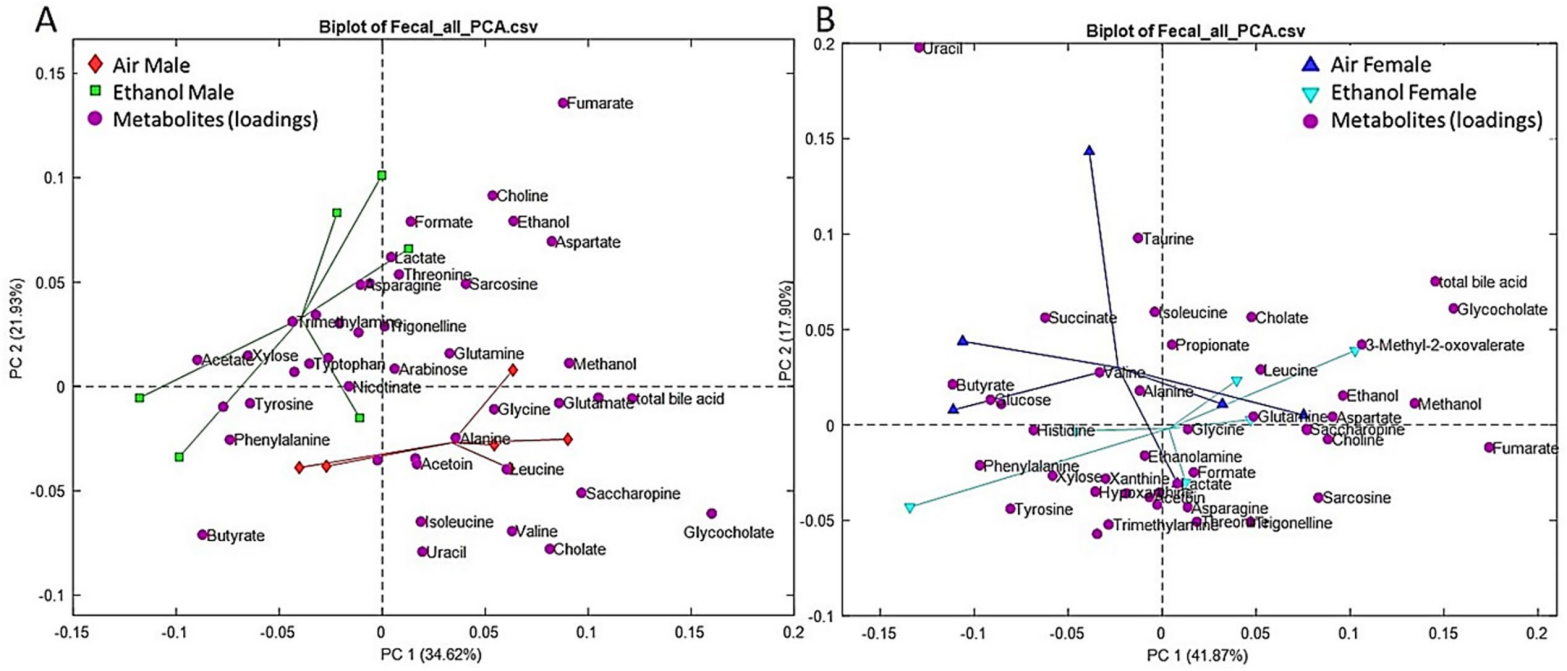
PCA of short-term withdrawal fecal metabolites. **(A)** PCA results from male fecal samples. Red diamonds represent male AIR and green squares represent male AIE. **(B)** PCA results from female fecal samples. Blue upward triangles represent female AIR and cyan downward triangles represent female AIE. Magenta circles (both panels) represent metabolite loadings (contributions to the PCA model).

**Figure 6:**
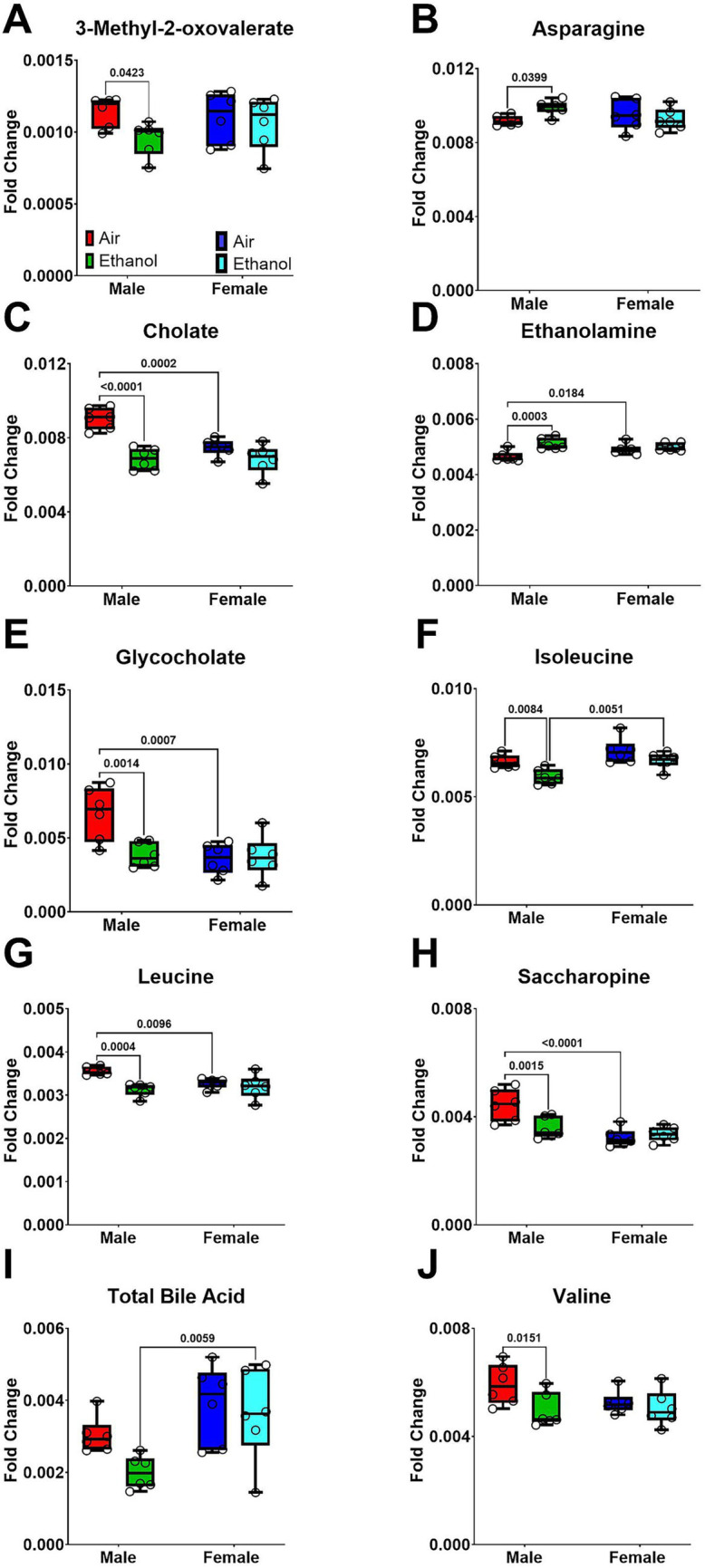
Selected fecal metabolites during short-term withdrawal following AIE. Samples include male AIR (red), male AIE (green), female AIR (blue), and female AIE (cyan). Data are represented as box-and-whisker plots showing median and range. All data points are shown as open circles. **(A)** 3-Methyl-2-oxovalerate; **(B)** Asparagine; **(C)** Cholate; **(D)** Ethanolamine; **(E)** Glycocholate; **(F)** Isoleucine; **(G)** Leucine; **(H)** Saccharopine; **(I)** Total Bile Acid; **(J)** Valine.

Short-term withdrawal fecal samples were collected between PND 49–51, which was approximately 1 week after the last cycle of AIE exposure. Metabolic profiling showed distinct differences between AIR and AIE male mice (Figure 5A). In contrast, female AIR and AIE mice showed only subtle differences in metabolic profiles (Figure 5B). Metabolites showing significant changes after exposure or between sexes are presented in Figures 6A–J. Primary metabolites contributing to separation in male AIE and AIR mice included amino acids such as isoleucine [Exposure (F_1, 20_ = 11.36, *p* < 0.004); Sex (F_1, 20_ = 13.54, *p* < 0.002); Figure 6F] and leucine [Sex by Exposure (F_1, 20_ = 6.26, *p* < 0.03); Exposure (F_1, 20_ = 12.45, *p* < 0.003); Figure 6G]. Additional amino acids that contributed to AIE-related separation in males included asparagine [Sex by Exposure (F_1, 20_ = 4.53, *p* < 0.05); Figure 6B] and valine [Exposure (F_1, 20_ = 5.18, *p* < 0.04); Figure 6J]. Other metabolites that contributed to group separations included cholate [Sex by Exposure (F_1, 20_ = 10.06; *p* < 0.005); Sex (F_1, 20_ = 10.06, *p* < 0.005); Exposure (F_1, 20_ = 32.27, *p* < 0.001); Figure 6C], ethanolamine [Sex by Exposure (F_1, 20_ = 6.56, *p* < 0.02); Exposure (F_1, 20_ = 12.50, *p* < 0.003); Figure 6D], glycocholate [Sex by Exposure (F_1, 20_ = 7.68, *p* < 0.02); Sex (F_1, 20_ = 8.37, *p* < 0.01); Exposure (F_1, 20_ = 6.15, *p* < 0.03); Figure 6E], and saccharopine [Sex by Exposure (F_1, 20_ = 8.96, *p* < 0.01); Sex (*F* (1, 20) = 17.97, *p* < 0.0005); Exposure (F_1, 20_ = 4.77, *p* < 0.05); Figure 6H].

#### 3.3.3 Fecal metabolites: long-term withdrawal

**Figure 7:**
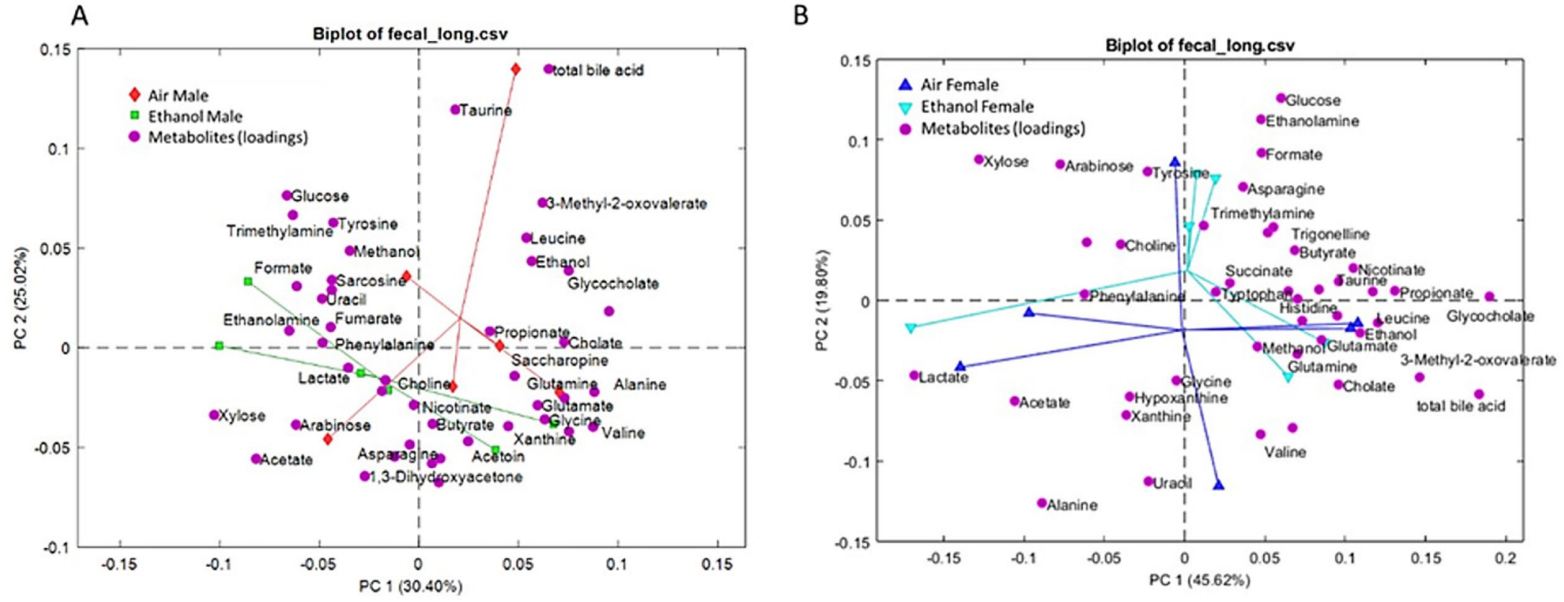
PCA of long-term withdrawal fecal metabolites. **(A)** PCA results from male fecal samples. Red diamonds represent male AIR and green squares represent male AIE. **(B)** PCA results from female fecal samples. Blue upward triangles represent female AIR and cyan downward triangles represent female AIE. Magenta circles (both panels) represent metabolite loadings (contributions to the PCA model).

**Figure 8:**
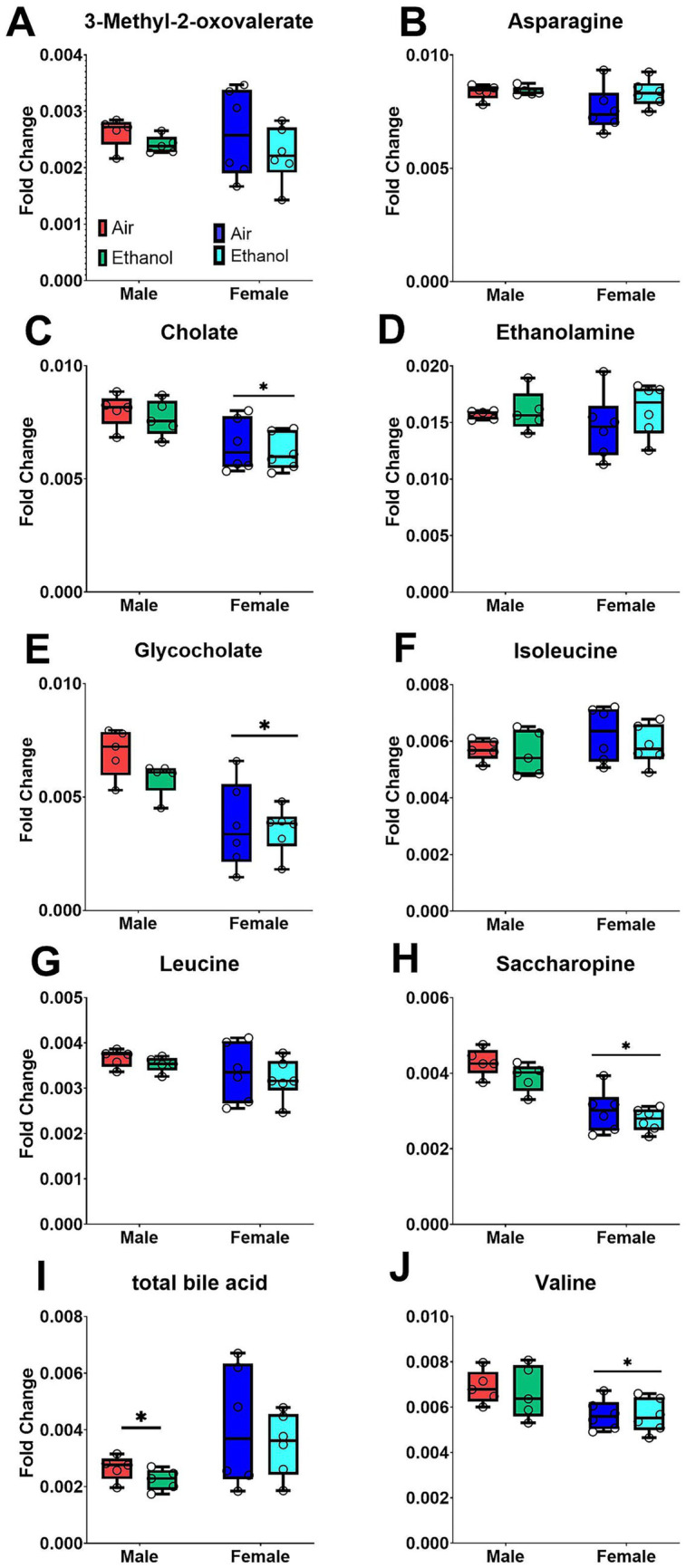
Selected fecal metabolites during long-term withdrawal following AIE. Samples include male AIR (red), male AIE (green), female AIR (blue), and female AIE (cyan). Data are represented as box-and-whisker plots showing median and range. All data points are shown as open circles. **(A)** 3-Methyl-2-oxovalerate; **(B)** Asparagine; **(C)** Cholate; **(D)** Ethanolamine; **(E)** Glycocholate; **(F)** Isoleucine; **(G)** Leucine; **(H)** Saccharopine; **(I)** Total Bile Acid; **(J)** Valine.

For long-term withdrawal, fecal samples were collected between PND 91–95 (Figure 7). Male samples showed partial separation between AIE and AIR groups (Figure 7A), whereas female samples showed greater overlap in the metabolic profiles between AIR and AIE mice (Figure 7B). Metabolites showing significant changes after exposure or between sexes are presented in Figures 8A–J. Sex differences in overall metabolic profiles regardless of exposure for cholate [Sex (F_1, 18_ = 15.37, *p* < 0.0002); Figure 8C], glycocholate [Sex (F_1, 18_ = 25.10, *p* < 0.0001); Figure 8E], saccharopine [Sex (F_1, 18_ = 45.00, *p* < 0.0001), Figure 8H], total bile acid [Sex (F_1, 18_ = 5.99, *p* < 0.03), Figure 8I], and valine [Sex (F_1, 18_ = 9.69, *p* < 0.01), Figure 8J]. Overall, AIE exposure did not produce clear separation of fecal metabolite profiles in male or female mice at long-term withdrawal.

#### 3.3.4 Liver metabolites

**Figure 9:**
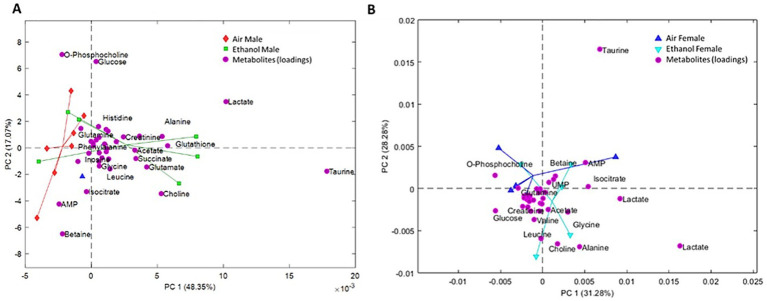
PCA of liver metabolites. **(A)** PCA results from male liver samples. Red diamonds represent male AIR and green squares represent male AIE. **(B)** PCA results from female liver samples. Blue upward triangles represent female AIR and cyan downward triangles represent female AIE. Magenta circles (both panels) represent metabolite loadings (contributions to the PCA model).

**Figure 10:**
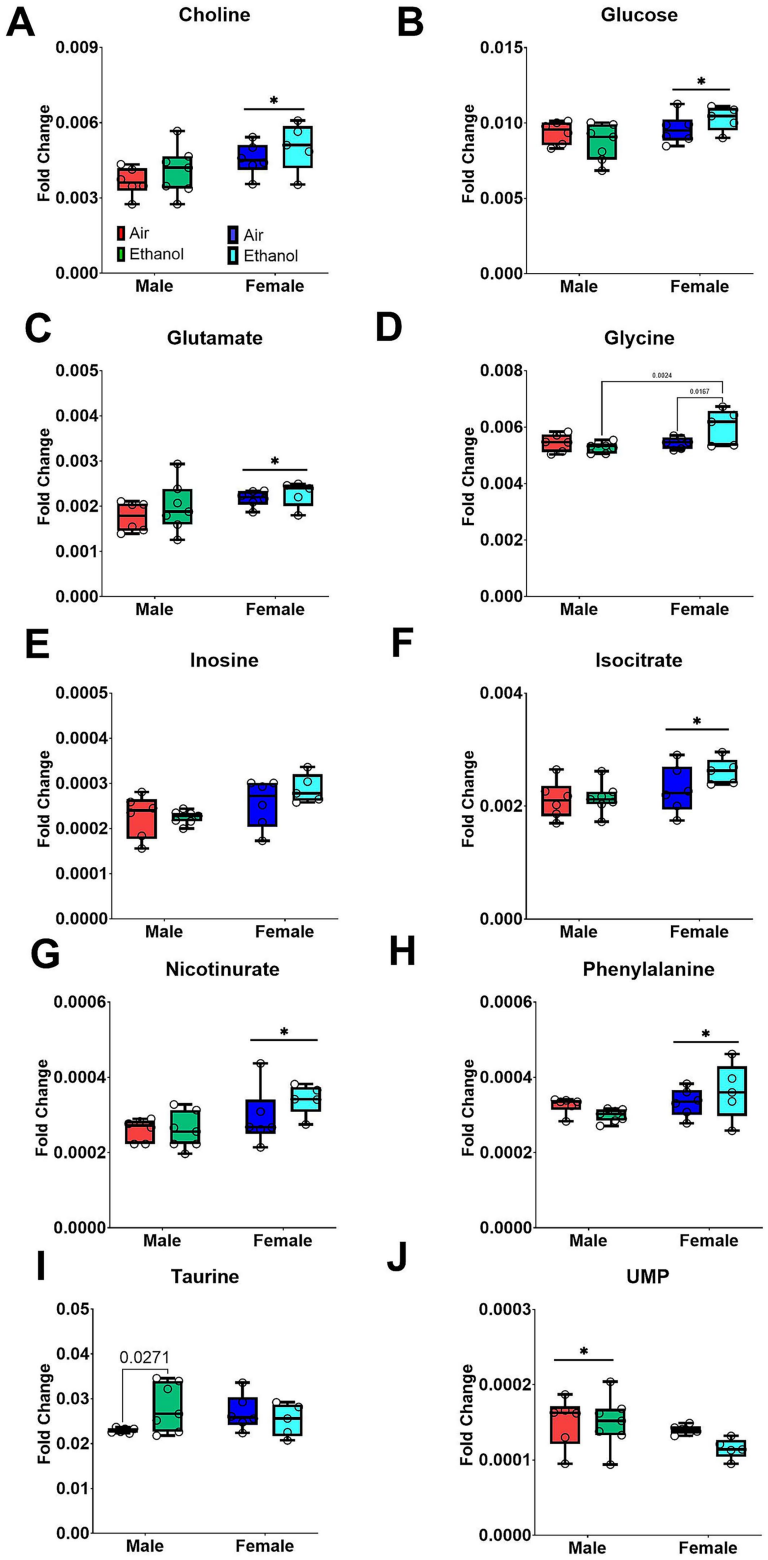
Selected liver metabolites. Samples include male AIR (red), male AIE (green), female AIR (blue), and female AIE (cyan). Data are represented as box-and-whisker plots showing median and range. All data points are shown as open circles. **(A)** Choline; **(B)** Glucose; **(C)** Glutamate; **(D)** Glycine; **(E)** Inosine; **(F)** Isocitrate; **(G)** Nicotinurate; **(H)** Phenylalanine; **(I)** Taurine; **(J)** UMP.

Three days following the LORR test (detailed below), liver samples were collected on PND 119. Importantly, all mice received the ethanol challenge used for the LORR test prior to liver sample collection and subsequent metabolomic analyses. Liver metabolites did not show clear separation in the PCA score plots for either male or female samples, suggesting no major AIE-related perturbation in liver metabolite profiles. Although male and female samples remain distinguishable, AIR and AIE groups showed only partial separation (Figure 9). Metabolites showing significant changes after exposure or between sexes are presented in Figures 10A–J.

Most differences in liver metabolite profiles reflected sex differences between males and females, regardless of exposure including choline [Sex (F_1, 20_ = 7.81, *p* < 0.02); Figure 10A], glucose [Sex (F_1, 20_ = 5.16, *p* < 0.05); Figure 10B], glutamate [Sex (F_1, 20_ = 4.961, *p* < 0.05); Figure 10C], isocitrate [Sex (F_1, 20_ = 6.02, *p* < 0.03); Figure 10F], nicotinurate [Sex (F_1, 20_ = 6.02, *p* < 0.03); Figure 10G], phenylalanine [Sex (F_1, 20_ = 4.40, *p* < 0.05); Figure 10H], and UMP [Sex (F_1, 20_ = 4.69, *p* < 0.05); Figure 10J]. Female AIE mice had higher glycine levels compared to Female AIR mice and their Male AIE counterparts [Sex by Exposure (F_1, 20_ = 6.06, *p* < 0.03); Sex (F_1, 20_ = 6.16, *p* < 0.03); Figure 10D]. In contrast, ethanol increased taurine levels only in Male AIE mice compared to Male AIR mice [Sex by Exposure (F_1, 20_ = 4.56, *p* < 0.05); Figure 10I].

### 3.4 Loss of righting reflex

**Figure 11:**
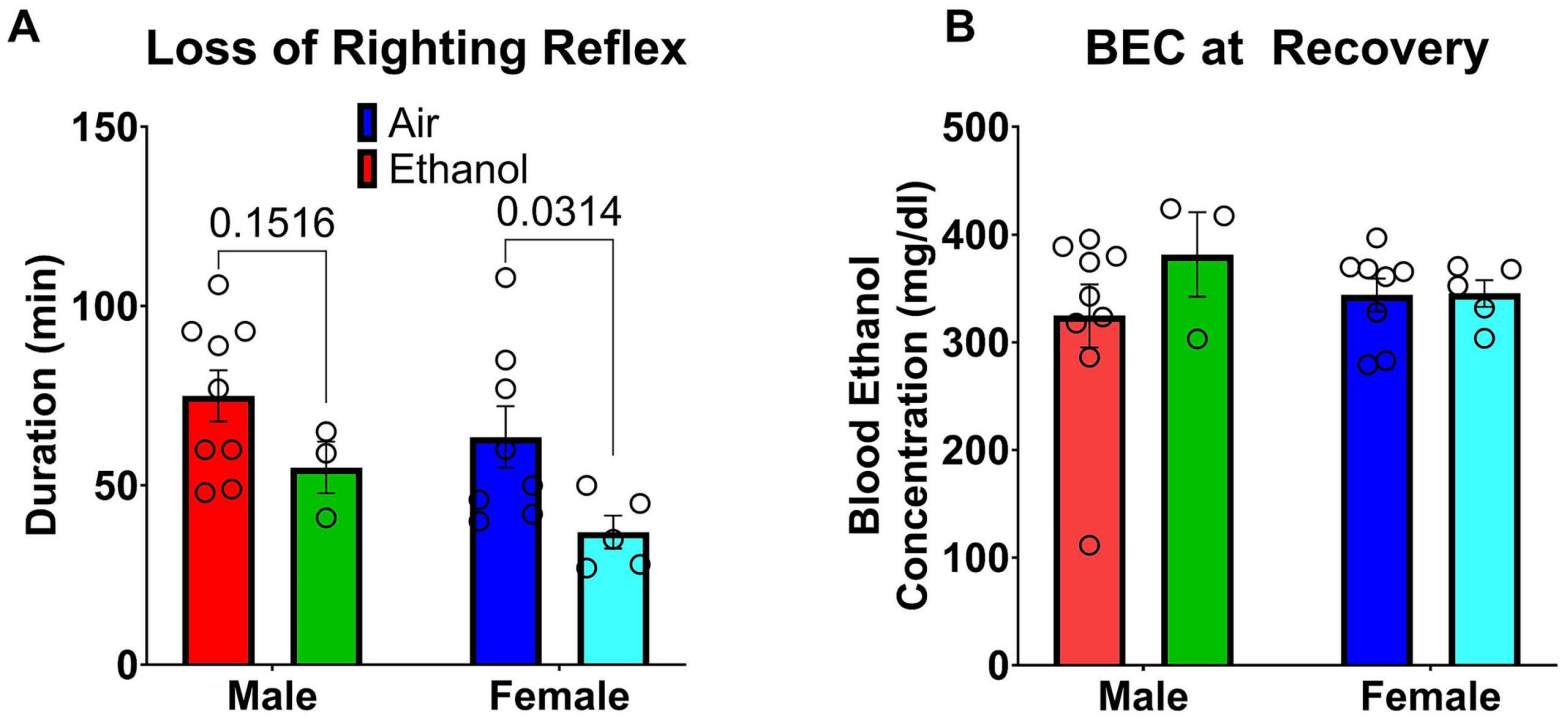
Loss of righting reflex. **(A)** Duration of loss of righting reflex (min). **(B)** Blood ethanol concentration (BEC) at recovery of loss of righting reflex (mg/dL). All data are shown as mean ± SEM.

On PND 116, all mice were assessed for LORR, and BEC was measured upon recovery for mice that exhibited LORR (Figure 11). LORR duration and recovery BEC analyses include only mice that exhibited LORR. Among mice that exhibited LORR, AIE-exposed mice showed shorter LORR duration than AIR-exposed mice [Exposure (F_1, 21_ = 6.91, *p* < 0.02)], an effect that was most evident in female mice (Figure 11A). However, there were no differences in BEC at recovery between groups regardless of adolescent AIR or AIE exposure (Figure 11B). It is important to note that only 3 out of 10 Male-AIE mice lost their righting reflex compared to 9 out of 10 Male-AIR mice lost their righting reflex, and only 5 out of 10 Female-AIE mice lost their righting reflex compared to 8 out of 10 Female-AIR mice. Consistent with reduced sensitivity to ethanol’s sedative effects, AIE reduced LORR incidence in males (3/10 vs. 9/10, Fisher’s exact *p* = 0.020), whereas the reduction in females (5/10 vs. 8/10) was not significant (*p* = 0.35). For mice that did not exhibit LORR, BECs were collected at a matched post-injection timepoint (when cage mates recovered) to confirm ethanol exposure. Matched-timepoint BECs were available for Male-AIE mice (*n* = 3; mean ± SEM = 378.9 ± 22.7 mg/dL), Male-AIR (*n* = 1; 318.1 mg/dL), and Female-AIR (*n* = 2; 420.1 ± 13.0 mg/dL). Matched-timepoint BECs were unavailable for Female-AIE mice that did not exhibit LORR (0/5).

## 4 Discussion

A growing body of research links excessive alcohol consumption to changes in the gut microbiome, which can directly contribute to health issues such as alcohol use disorder and alcohol-associated liver disease (Wolstenholme et al., 2024; Wang X. et al., 2023). In the present study, AIE vapor exposure models intermittent binge-like ethanol exposure during adolescence, rather than a model of established alcohol dependence or AUD. Therefore, we refer to AUD primarily as a translational context for interpreting metabolomic pathways and potential biomarkers. In addition, neuropsychiatric disorders can be caused by disruptions in the gut-brain axis (Wolstenholme et al., 2024). Metabolomics is also a newer methodological approach that supports biomarker identification in AUD and many other neuropsychiatric disorders (Harada et al., 2016). Specifically, cellular processes impacted by environmental stressors, such as repeated ethanol exposure, can be identified through metabolomic profiling. This includes noting alterations in amino acid, lipid, and carbohydrate metabolism (Harada et al., 2016; Harrigan et al., 2008).

Chronic alcohol exposure can disrupt the gut-liver and gut-brain axes by altering the complex relationship between the gut and the liver through various mechanisms (Wolstenholme et al., 2024). Gut dysbiosis and impaired gut-liver metabolism resulting from microbiome changes are linked to the advancement of alcohol-induced liver disease (Ganesan et al., 2024). Human studies that compare alcohol users and nonusers have found that alcohol consumption alters the metabolome profiles of lipids and amino acids, which are linked to energy metabolism (Voutilainen and Kärkkäinen, 2019; Clark et al., 2025). An adult male rat model of AUD showed changes in lipid, amino acid, nucleotide, and carbohydrate metabolism pathways in fecal samples (Wang X. et al., 2023).

Ethanol exposure has been associated with metabolites including lipoprotein and glucose (Du et al., 2020; Li et al., 2018; Wang X. et al., 2023). Binge ethanol exposure can hinder the molecular transport of albumin, transferrin, and glycoproteins, and lead to a decrease in protein synthesis, such as glycoprotein synthesis (Forman, 1988). The decreased levels of amino acids found in the serum samples from our study support this conclusion. Amino acids are not just markers, they are substrates. When circulating amino acid pools shift after ethanol exposure, it reflects disrupted protein synthesis and altered energy balance, especially in the liver, where protein turnover and metabolic demand are high (Harrigan et al., 2008; Voutilainen and Kärkkäinen, 2019). Changes in essential amino acids and branched-chain amino acids can signal changes in protein catabolism and fuel utilization, while shifts in methionine and threonine implicate methyl-donor and redox pathways that support glutathione-dependent antioxidant defenses (Harada et al., 2016; Harrigan et al., 2008).

Oxidative stress and alterations in amino acid and energy metabolism have been implicated in alcohol-related physiological stress and neuropsychiatric outcomes (Humer et al., 2020a; Humer et al., 2020b). However, because exposure-related effects on anxiety-like behavior were modest and not uniformly detected across assays in the present study, we do not interpret these metabolite shifts as direct correlates of anxiety-like behavior. Our findings align with previous research, as we also observed alterations in numerous amino acid metabolites including leucine, isoleucine, tryptophan, tyrosine, threonine, and methionine levels.

The open field test is a common measure of anxiety-like behavior in mice. This test utilizes the center zone as a measure of thigmotaxis; due to their natural aversion to bright open spaces, mice that display increased anxiety tend to avoid the center (Bolivar et al., 2000; Bailey and Crawley, 2009; Lipkind et al., 2004; Simon et al., 1994; Walz et al., 2016). Our research demonstrated that adult male mice exposed to ethanol during adolescence spent more time in the center zone and reared more than air-exposed males, a pattern consistent with reduced thigmotaxis in the OFT. Females did not show an exposure-related difference in the OFT, indicating that this effect was male-specific in the present data. However, the light/dark test did not detect exposure-related group differences. In the LDT, the only consistent pattern was a sex difference in latency to enter the light compartment (males longer than females), independent of exposure. These data indicate that AIE-related effects on affect-related behavior are limited and depend on sex and withdrawal timepoint (Peeters et al., 2024).

In the tail suspension test, AIE exposure was associated with longer latency to become immobile in female mice compared to AIR controls, and this exposure effect was absent in males. There were no exposure-related differences in duration of immobility at either withdrawal time point. However, both males and females spent more time immobile during long-term withdrawal compared to short-term withdrawal.

In adulthood, AIE exposure also reduced sensitivity during the LORR challenge. AIE reduced the proportion of mice that exhibited LORR (most evident in males) and shortened LORR duration among mice that did exhibit LORR (most evident in females). These findings are consistent with reduced sensitivity to ethanol’s sedative effects and a tolerance-like shift in the dose required to produce LORR.

A human study conducted by Leclercq et al. (2024). investigated the relationship between affective behaviors (anxiety, depression, and alcohol craving) and metabolomic changes in patients with and without AUD. The researchers found that the metabolome profiles of patients with AUD differed from those without AUD. Additionally, in some cases, abstinence led to a return to levels seen in healthy controls (Leclercq et al., 2024). Recent work has assessed similar changes induced by alcohol dependence in behavioral changes and changes in fecal and serum metabolite profiles after chronic intermittent two bottle choice drinking in adult Sprague Dawley rats. Specifically, these rats were tested on the 28th day of intermittent ethanol exposure (Wang X. et al., 2023). Wang X. et al. (2023) reported increased anxiety-like behavior in an adult male AUD rat model, with EtOH-exposed rats spending less time in the center of the open field and less time in the open arms of the elevated plus maze than controls. This direction differs from our modest increase in center time in male mice, which we interpret as a task-specific and withdrawal timepoint-dependent effect. Because Wang B. et al. (2023) and Wang X. et al. (2023) examined adult male rats, this comparison is specific to our male mouse outcomes. Our work showed moderate changes in anxiety-like behavior and correlations between metabolites in serum, fecal, and liver samples. Our work differed from previous research in several key ways. First, it included both male and female subjects. Second, it involved multiple fecal sample collections at different time points throughout the ethanol withdrawal process. Third, it examined changes in liver metabolites following AIE exposure and after an adult ethanol challenge (LORR) prior to tissue collection.

Costanzo et al. (2022) examined sex differences in plasma metabolomics in humans and found that men tended to have higher levels of amino acids than women. Baseline sex differences in metabolomic profiles are well documented (Costanzo et al., 2022; Krumsiek et al., 2015; Brennan and Gibbons, 2020). These patterns were also evident in our data, independent of exposure during adolescence. We interpret AIE-related effects primarily using within-sex comparisons (AIE vs. AIR) and Sex by Exposure patterns, rather than drawing exposure-related conclusions from sex main effects alone. In that study, these amino acids included phenylalanine, glutamate, glutamine, kynurenine, methionine, proline, and tyrosine. Additionally, men had higher levels of the branched-chain amino acids (BCAA) valine, leucine, and isoleucine (Costanzo et al., 2022). In human men, higher alcohol consumption was associated with levels of serum threonine, glutamine, and guanidinosuccinate (Harada et al., 2016). In serum samples, lipid, amino acid, and carbohydrate metabolism were differently affected by intermittent ethanol exposure in the adult male rats (Wang X. et al., 2023). Similar to recent work in adult male rats (Wang X. et al., 2023), we also found changes in metabolites in the ethanol-exposed group compared to the air-exposed group in serum and fecal samples, including proline, glycine, serine, tryptophan, lysine, and threonine. These metabolites are linked to pathways involved in oxidative stress and immune functions. The AIE-induced metabolite differences observed in male mice in the current study showed overlap with patterns reported in prior work conducted largely in males (e.g., Wang X. et al., 2023). Because baseline sex differences in metabolomic profiles are robust, we interpret female outcomes primarily using within-sex comparisons (AIE vs. AIR) rather than direct alignment with male-only studies.

Changes in epigenetic modifications, addiction, and reward networks in the brain are associated with alterations in specific metabolites, including butyrate (Wolstenholme et al., 2024). Other work in humans showed specific changes in the gut microbiome associated with AUD and alcoholic liver disease, including a decrease in butyrate-producing bacteria and an increase in endotoxin-producing bacteria (Litwinowicz and Gamian, 2023). In our current work, butyrate was associated with anxiety-like behavior in the short-term withdrawal fecal samples in male mice. Similar to our work, there were some distinct metabolites altered in serum compared to fecal samples after ethanol exposure (Wang X. et al., 2023).

Perturbations in metabolic processes can induce a greater susceptibility to developing AUD and associated disorders after prolonged use (Harrigan et al., 2008). Many of the deleterious effects of heavy alcohol use may be secondary to alcohol’s impact on liver function (Harrigan et al., 2008). To date there is limited research examining changes in liver metabolomic profiles in rodent models of heavy alcohol exposure. To our knowledge this is the first study to date to determine long-term changes in liver metabolic profiles following adolescent ethanol exposure in mice.

A potential limitation of this study was the use of NMR-based analysis, rather than LC/MS/MS techniques, which may have limited the identification of differentially expressed metabolites between groups (Voutilainen and Kärkkäinen, 2019; Yang et al., 2024). The different collection times and potential compensatory changes between sample types made it difficult to identify which metabolites were altered across withdrawal periods (Voutilainen and Kärkkäinen, 2019). Overall, our present work represents a semi-cross-sectional characterization of alcohol-induced metabolite profiles across serum, fecal, and liver samples collected at defined withdrawal time points after AIE exposure in male and female mice. We selected the short-term withdrawal behavioral window (PND 49 to 53) to align behavioral testing with fecal collection and minimize additional handling and stress, because fecal samples could be collected following the open field test without extra handling beyond the behavioral battery. We acknowledge that behavioral testing at a more acute withdrawal time point (e.g., 24 to 48 h) could potentially reveal additional or different behavioral effects, but this would have required additional handling and disruption of the animals. To capture more acute systemic effects of exposure, serum samples for metabolomics were collected 24 h after the final exposure cycle (PND 43).

The metabolome provides insight into functional changes and disease progression resulting from heavy alcohol use. Compared to other ‘omics’ approaches, the metabolome offers a deeper understanding of the overall functional alterations caused by metabolic processes (Voutilainen and Kärkkäinen, 2019). Several types of samples, including serum, urine, and fecal samples, can be used to test for metabolomic changes following heavy alcohol exposure including in humans and preclinical models (Harada et al., 2016; Harrigan et al., 2008; Wang X. et al., 2023; Yang et al., 2024). Targeted and nontargeted metabolomic approaches can be used to determine changes in metabolites that are altered after heavy alcohol exposure, with both having advantages and disadvantages (Harrigan et al., 2008). We employed a nontargeted metabolomic approach to comprehensively characterize the alterations caused by AIE vapor exposure during adolescence. This initial research aimed to establish a proof-of-concept model by analyzing how ethanol exposure during adolescence affects metabolic profiles in male and female mice. This was achieved by examining various sample types, including serum, fecal, and liver samples.

Metabolomics approaches can serve to help in treating afflicted individuals of AUD with a more personalized or tailored approach (Harrigan et al., 2008). Goffredo et al. (2017) found that obese adolescents with NAFLD have higher plasma levels of certain amino acids (valine, isoleucine, tryptophan, and lysine) compared to those without NAFLD. These elevated amino acid levels were associated with decreased insulin sensitivity and predicted an increase in hepatic fat content over time, independent of obesity and insulin resistance. These data together with those in the present work examining changes in affective behaviors, metabolite profiles in several different sample types following AIE, and incorporating sex as a variable can inform future work aimed at identifying specific metabolic biomarkers important for AUD progression.

## Data Availability

The raw data supporting the conclusions of this article will be made available by the authors, without undue reservation.
