# Supplementary material for: Adolescent intermittent ethanol exposure induces sex-specific and time-dependent changes in affective behaviors and metabolomic profiles
Source: Front Behav Neurosci. 2026 Jan 30;19:1614537. doi: 10.3389/fnbeh.2025.1614537 (PMC12901355; doi:10.3389/fnbeh.2025.1614537)
Supplement: Supplementary file 1 [file Table_1.docx]

Supplemental Data: *Metabolomics: Activity relationship with serum, fecal, and liver samples*

# **1.1 Serum samples**

The correlation relationship of serum metabolites with the activities data were analyzed using the Pearson correlation relationship study. They are shown in **Table 6** below. The serum samples were collected on PND 43.

**Table S1: Correlation between behavior measures and serum metabolites**

|  | **Air Male** | | **EtOH Male** | | **Air Female** | | **EtOH Female** | |
| --- | --- | --- | --- | --- | --- | --- | --- | --- |
| **Alanine** |  |  | TDM  Center Zone Entries  Center Zone TDM | r = 0.83, p = 0.041  r = 0.89, p = 0.01  r = 0.86, p = 0.02 | Center Zone Time | r = -0.87, p = 0.02 |  |  |
| **Citrate** |  |  | Center Zone Time | r = -0.83, p = 0.03 |  |  |  |  |
| **Glucose** |  |  | TDM  Center Zone TIme Center Zone Entries  Center Zone TDM | r = -0.95, p = 0.003  r = -0.95, p = 0.003  r = -0.90, p = 0.01  r = 0.98, p = 0.0005 |  |  |  |  |
| **Glutamate** |  |  | Center Zone Time | r = -0.95, p = 0.002 | Center Zone Time | r = -0.83, p = 0.04 |  |  |
| **Glutamine** | LDT Time | r = -0.83, p = 0.03 | Center Zone Time | r = -0.92, p = 0.009 |  |  | Rearing | r = 0.97, p = 0.001 |
| **Isoleucine** |  |  | LDT Time | r = 0.85, p = 0.02 |  |  | TDM  Center Zone Entries  Center Zone TDM | r = 0.83, p = 0.03  r = 0.91, p = 0.01  r = 0.86, p = 0.02 |
| **Lactate** |  |  |  |  | Center Zone Time  Center Zone Entries  Center Zone TDM | r = 0.87, p = 0.02  r = 0.87, p = 0.02  r = 0.89, p = 0.01 |  |  |
| **Leucine** |  |  | Center Zone TDM | r = 0.84, p = 0.03 | LDT Time | r = -0.92, p = 0.008 |  |  |
| **Lipoprotein** |  |  | TDM  Center Zone Time  Center Zone Entries  Center Zone TDM | r = 0.85, p = 0.03  r = 0.98, p = 0.006  r = 0.82, p = 0.046  r = 0.88, p = 0.01 |  |  |  |  |
| **Phenylalanine** |  |  | Center Zone Time  Center Zone TDM | r = -0.90, p = 0.01  r = -0.83, p = 0.03 |  |  |  |  |
| **Proline** | TDM  Center Zone Entries  Center Zone TDM | r = 0.83, p = 0.04  r = 0.83, p = 0.041  r = 0.83, p = 0.042 | Center Zone Time  Center Zone TDM | r = -0.87, p = 0.02  r = 0.84, p = 0.03 | TDM  Rearing  Center Zone Entries  Center Zone TDM | r = -0.85, p = 0.03  r = -0.96, p = 0.002  r = -0.89, p = 0.01  r = -0.87, p = 0.02 |  |  |
| **Succinate** |  |  | Center Zone Time  Center Zone TDM | r = -0.94, p = 0.004  r = -0.84, p = 0.03 |  |  |  |  |
| **sn-Glycero-3-phosphocholine** |  |  | TDM  Center Zone TDM | r = 0.91, p = 0.01  r = 0.89, p = 0.01 |  |  |  |  |
| **Taurine** |  |  | Center Zone Time | r = -0.85, p = 0.03 |  |  |  |  |
| **Tyrosine** |  |  | LDT Time | r = -0.83, p = 0.03 |  |  |  |  |

**1.2 Fecal samples short-term withdrawal**

The correlation relationship of early withdrawal fecal metabolites with the activities data were analyzed using the Pearson correlation relationship study. They are shown in **Table 7** below. The fecal samples were collected between PND 49-53.

**Table S2: Correlation between behavior measures and short-term withdrawal fecal metabolites**

|  | **Air Male** | | **EtOH Male** | | **Air Female** | | **EtOH Female** | |
| --- | --- | --- | --- | --- | --- | --- | --- | --- |
| **1,3-Dihydroxyacetone** | TDM  Center Zone Time  Center Zone Entries  Center Zone TDM | r = 0.94; p = 0.004  r = 0.85; p = 0.03  r = 0.93; p = 0.005  r = 0.93; p = 0.007 | TDM  Center zone TDM | r = -0.87; p = 0.02  r = -0.82; p = 0.048 | LDT Time | r = 0.83; p = 0.041 |  |  |
| **3-Methyl-2-oxovalerate** | TDM  Center Zone Time  Center Zone Entries  Center Zone TDM | r = -0.93; p = 0.007  r = -0.81; p = 0.049  r = 0.95; p = 0.003  r = -0.91; p = 0.01 |  |  |  |  |  |  |
| **Acetoin** |  |  |  |  | LDT Time | r = 0.81; p = 0.049 |  |  |
| **Alanine** |  |  |  |  | LDT Time | r = -0.94; p = 0.005 |  |  |
| **Asparagine** |  |  | Center zone TDM  LDT Time | r = -0.82; p = 0.045  r = -0.92; p = 0.008 |  |  |  |  |
| **Aspartate** | Center zone time | r = -0.87; p = 0.02 | TDM  Center zone time  Center zone TDM | r = -0.88; p = 0.02  r = -0.83; p = 0.04  r = -0.86; p = 0.02 |  |  |  |  |
| **Butyrate** | TDM    Center zone time  Center Zone Entries  Center Zone | r = -0.93; p = 0.006  r = 0.83; p = 0.04  r = -0.82; p = 0.004  r = 0.91; p = 0.01 | LDT Time | r = 0.84; p = 0.03 |  |  |  |  |
| **Formate** |  |  | Center zone time | r = -0.82; p = 0.046 | LDT Time | r = 0.92; p = 0.009 | Center zone time  Center zone entries  Center zone TDM | r = 0.84; p = 0.03  r = 0.91; p = 0.01  r = 0.89; p = 0.01 |
| **Fumarate** | Center Zone Entries | r = -0.83; p = 0.04 |  |  |  |  |  |  |
| **Glucose** |  |  | TDM | R = 0.90; p = 0.01 |  |  |  |  |
| **Glutamate** | TDM | r = -0.83; p = 0.04 |  |  |  |  |  |  |
| **Glycocholate** | TDM  Center Zone Entries  Center Zone TDM | r = -0.84; p = 0.04  r = -0.84; p = 0.036  r = -0.81; p = 0.048 |  |  |  |  | Center zone time  Center zone TDM | r = 0.88; p = 0.02  r = 0.86; p = 0.02 |
| **Hypoxanthine** | TDM  Center Zone TDM | r = -0.81; p = 0.049  r = 0.82; p = 0.04 |  |  |  |  |  |  |
| **Lactate** | LDT Time | r = -0.83; p = 0.04 | TDM  Center zone TDM LDT Time | r = -0.85; p = 0.03  r = -0.89; p = 0.01  r = -0.84; p = 0.03 | LDT Time | r = 0.88; p = 0.02 |  |  |
| **Leucine** |  |  |  |  |  |  | Center zone time | r = 0.81; p = 0.049 |
| **Nicotinate** | TDM  Center Zone Time  Center Zone TDM | r = 0.99; p = 0.0001  r = 0.89; p = 0.017  r = 0.99; p = 0.0002 |  |  | LDT Time | r = 0.84; p = 0.03 | Center zone time  Center zone TDM | r = -0.84; p = 0.03  r = -0.88; p = 0.01 |
| **Phenylalanine** | TDM  Center Zone  Time  Center Zone Entries Center Zone TDM1 | r = 0.86; p = 0.03  r = 0.97; p = 0.001  r = 0.87; p = 0.02  r = 0.90; p = 0.01 |  |  |  |  | Center zone time Center zone TDM | r = -0.82; p = 0.044  r = -0.84; p = 0.03 |
| **Saccharopine** | TDM | r = -0.82; p = 0.048 |  |  |  |  |  |  |
| **Succinate** | Rearing | r = 0.93; p = 0.007 |  |  |  |  |  |  |
| **Taurine** |  |  |  |  |  |  | TDM | r = 0.85; p = 0.03 |
| **Threonine** |  |  | TDM  Center zone time  Center zone entries Center zone TDM | r = -0.99; p = 0.0002  r = -0.84; p = 0.03  r = -0.93; p = 0.006  r = -0.96; p = 0.002 | LDT Time | r = 0.86; p = 0.03 |  |  |
| **Total Bile Acid** |  |  | Center zone time | r = -0.86; p = 0.02 |  |  |  |  |
| **Trimethylamine** |  |  | Rearing | r = 0.82; p = 0.045 |  |  |  |  |
| **Tryptophan** |  |  |  |  | LDT Time | r = 0.91; p = 0.01 | Rearing | r = -0.98; p = 0.0007 |
| **Tyrosine** |  |  |  |  |  |  | TDM | r = -0.88; p = 0.01 |

#

# **1.3 Fecal samples long-term withdrawal**

The correlation relationship of late withdrawal fecal metabolites with the activities data were analyzed using the Pearson correlation relationship study. They are shown in **Table 8** below. The fecal samples were collected between PND 91-95.

**Table S3: Correlation between behavior measures and long-term withdrawal fecal metabolites**

|  | **Air Male** | | **EtOH Male** | | **Air Female** | | **EtOH Female** | |
| --- | --- | --- | --- | --- | --- | --- | --- | --- |
| **1,3-Dihydroxyaceton** | TDM  Center Zone TDM | r = -0.93; p = 0.01  r = -0.93; p = 0.01 |  |  |  |  |  |  |
| **Asparagine** |  |  |  |  | Rearing | r = 0.82; p = 0.04 |  |  |
| **Aspartate** | LDT Time | r = -0.90; p = 0.03 |  |  |  |  |  |  |
| **Choline** |  |  | Center Zone Time | r = 0.92; p = 0.02 |  |  |  |  |
| **Formate** | Center Zone Time  LDT Time | r = 0.93; p = 0.02  r = 0.95; p = 0.01 |  |  |  |  |  |  |
| **Glucose** | Center Zone Time  LDT Time | r = -0.92; p = 0.02  r = -0.93; p = 0.01 | LDT Time | r = 0.89; p = 0.04 |  |  |  |  |
| **Glutamine** | Center Zone Time | r = 0.99; p = 0.001 |  |  |  |  |  |  |
| **Hypoxanthine** | Center Zone Entries | r = 0.93; p = 0.01 |  |  |  |  |  |  |
| **Nicotinate** |  |  |  |  |  |  | Center Zone TIme | r = 0.82; p = 0.04 |
| **Phenylalanine** | Rearing  LDT Time | r = 0.98; p = 0.002  r = -0.90; p = 0.03 |  |  | Center Zone Time  Center Zone Entries | r = 0.94; p = 0.004  r = 0.88; p = 0.02 |  |  |
| **Proprionate** |  |  | Rearing | r = -0.94; p = 0.01 |  |  | LDT Time | r = 0.81; p = 0.047 |
| **Saccharopine** |  |  |  |  |  |  | LDT Time | r = 0.93; p = 0.006 |
| **Succinate** | LDT Time | r = 0.95; p = 0.01 |  |  |  |  |  |  |
| **Taurine** |  |  |  |  |  |  | Rearing | r = 0.89; p = 0.01 |
| **Threonine** |  |  | LDT Time | r = -0.91; p = 0.02 |  |  | LDT Time | r = 0.81; p = 0.047 |
| **Trimethylamine** | LDT Time | r = 0.96; p = 0.007 |  |  | TDM  Center Zone Entries  Center Zone TDM | r = 0.87; p = 0.02  r = 0.87; p = 0.02  r = 0.87; p = 0.02 |  |  |
| **Typtophan** |  |  |  |  | TDM  Center Zone Entries  Center Zone TDM | r = 0.81; p = 0.05  r = 0.95; p = 0.003  r = 0.81; p = 0.05 | Center Zone Time | r = 0.85; p = 0.03 |
| **Tyrosine** | Rearing  LDT Time | r = 0.94; p = 0.01  r = 0.96; p = 0.008 |  |  | Rearing  Center Zone Entries | r = 0.87; p = 0.02  r = 0.92; p = 0.009 |  |  |
| **Xanthine** | Center Zone Time | r = 0.90; p = 0.03 |  |  | Center Zone TIme | r = 0.81; p = 0.04 |  |  |

**1.4 Liver samples long-term withdrawal**

The correlation relationship of liver metabolites with the activities data from the last behavioral trial were analyzed using the Pearson correlation relationship study. They are shown in **Table 9** below. The liver samples were collected on PND 119.

**Table S4: Correlation between behavior measures and liver metabolites**

|  | **Air Male** | | **EtOH Male** | | **Air Female** | | **EtOH Female** | |
| --- | --- | --- | --- | --- | --- | --- | --- | --- |
| **Acetate** | Rearing | r = 0.82; p = 0.041 |  |  |  |  |  |  |
| **Betaine** | Center Zone TDM | r = -0.86; p = 0.02 |  |  |  |  |  |  |
| **Choline** | Rearing | r = 0.93; p = 0.007 |  |  |  |  |  |  |
| **Creatinine** |  |  | Center Zone Entries  Center Zone TDM | r = 0.81; p = 0.02  r = 0.78; p = 0.03 |  |  |  |  |
| **Glutamate** | Rearing | r = 0.82; p = 0.043 | Center Zone Entries  Center Zone TDM | r = 0.79; p = 0.03  r = 0.78; p = 0.03 |  |  |  |  |
| **Glycine** |  |  |  |  | LDT Time | r = 0.89; p = 0.03 |  |  |
| **Inosine** |  |  | Center Zone Entries  Center Zone TDM | r = -0.88; p = 0.008  r = -0.88; p = 0.008 |  |  |  |  |
| **Isoleucine** | Rearing | r = 0.84; p = 0.03 |  |  |  |  |  |  |
| **Isocitrate** |  |  | LDT Time | r = 0.91; p = 0.004 |  |  |  |  |
| **Lactate** | Center Zone TDM | r = 0.82; p = 0.043 |  |  |  |  |  |  |
| **Leucine** | Rearing | r = 0.94; p = 0.004 |  |  |  |  |  |  |
| **O-phosphocholine** |  |  | Rearing | r = -0.83; p = 0.01 |  |  |  |  |
| **Phenylalanine** |  |  | TDM  Center Zone Entries | r = 0.78; p = 0.03  r = 0.75; p = 0.048 |  |  |  |  |
| **Pyruvate** |  |  | Center Zone Entries | r = 0.78; p = 0.03 |  |  |  |  |
| **Taurine** | Center Zone Time | r = 0.92; p = 0.008 |  |  | Rearing | r = -0.82; p = 0.041 |  |  |
| **Tyrosine** |  |  | Center Zone Entries  Center Zone TDM | r = 0.80; p = 0.02  r = 0.78; p = 0.03 |  |  |  |  |
| **UDP-galactose** |  |  | LDT Time | r = 0.77; p = 0.04 |  |  |  |  |
